# Is Prednisolone Useful in Treatment of Hyperemesis Gravidarum?

**DOI:** 10.7759/cureus.11128

**Published:** 2020-10-24

**Authors:** Aroosha Asmat, Iqra Yasin, Iffat Hamid, Riffat Nawaz

**Affiliations:** 1 Obstetrics & Gynecology, Sharif Medical & Dental College / Sharif Medical City Hospital, Lahore, PAK; 2 Obstetrics & Gynecology, Shalamar Medical & Dental College, Lahore, PAK

**Keywords:** first trimester, pregnancy, hyperemesis gravidarum, prednisolone, placebo

## Abstract

Objective: To compare the outcome of prednisolone versus placebo in females presenting with hyperemesis gravidarum during first trimester of pregnancy.

Methods: Randomized controlled trial was conducted in the Department of Obstetrics & Gynecology of Jinnah Hospital, Lahore over a period of 12 months. A total of 300 patients were divided equally into two groups, i.e. prednisolone 20 mg orally 12 hourly for seven days and placebo for the same duration. Follow up in the outpatient department after one week was done to assess for continuous and/or high-frequency vomiting (as per operational definitions). Statistical Package for Social Sciences (SPSS) Statistics version 22 (IBM Corp., Armonk, NY, USA) was used to analyze data. Quantitative and qualitative variables were assessed in terms of mean/standard deviation and frequency/percentage respectively. Comparison of two study groups and stratified confounding factors (age and parity) were assessed by chi-square test (significant P-valve ≤ 0.05).

Results: 28.7% of cases of the prednisolone group had continuous and/or high frequency vomiting in comparison to 46% of the placebo group (p=0.002).

Conclusions: Prednisolone is effective for treating women with hyperemesis gravidarum during the first trimester of pregnancy.

## Introduction

Hyperemesis gravidarum (HG) is defined as pernicious vomiting in pregnancy that requires hospitalization. It typically is associated with dehydration, electrolyte disturbances, and starvation with consequent weight loss [1,2]. Most women (70 to 80%) experience morning sickness (nausea) during pregnancy. This condition is generally harmless, and while morning sickness can be quite uncomfortable, it typically subsides within 12 weeks. HG is an extreme form of morning sickness that causes severe nausea and vomiting during pregnancy [3].

Nausea and vomiting begin in the first trimester, at about six to eight weeks’ gestation, typically peaking at about nine weeks’ gestation and settling by about 12 weeks [4]. Adequate oral hydration and avoidance of dietary triggers are often sufficient, but a proportion of women with severe and protracted nausea and vomiting will need antiemetic drugs. Women should have regular medical follow-ups to ensure steroids are not taken for lengthy periods [5].

One study reported that the rate of continuous vomiting (≥1 time / 24 hours) after one week was 41.7% with prednisolone and 58.3% with placebo [6]. There were 41.7% females with placebo who had vomiting >5 times/day while 16.7% was observed with prednisolone. Although the rate was very different, the difference was insignificant (P=0.26).

The rationale of this study was to compare the outcome of prednisolone with placebo in females presenting with HG during the first trimester of pregnancy. In literature, the beneficial effect of prednisolone for HG is mentioned but scarce data with limited sample sizes is available. Moreover, some studies such as mentioned above show an insignificant role of prednisolone in HG. Besides this, a lack of local studies in the Asian population also supports the need for study to resolve the controversial role of prednisolone with a large sample size.

## Materials and methods

A randomized controlled trial (RCT) was performed at Department of Obstetrics & Gynecology, Jinnah Hospital, Lahore over a period of 12 months from 05-12-2015 to 04-12-2016. Using 80% power of test and 95% confidence level, a sample size of 300 (150 each group) was calculated. Sampling technique of non-probability consecutive was used.

All pregnant women <13 weeks of gestation (as per last menstrual period - LMP) presenting with hyperemesis gravidarum (defined as ≥5 episodes of vomiting per day, for ≥1week in first trimester i.e. <13 weeks of gestational assessed on LMP). Women with a history of taking steroids in the last two months, proven peptic ulceration requiring treatment in the previous five years, overt thyrotoxicosis (T4 >160 nmol/L, T3 >2.7 nmol/L) were excluded from the study.

Approval of the hospital ethical committee was followed by recruitment via the obstetrics clinic. Detailed history and clinical examination was done. Random number tables were used to divide the cases into two equal groups, Group A: Prednisolone 20 mg orally 12 hourly for seven days and Group B: Placebo with the same dose regimen. It was hypothesized that there was a difference in outcome of prednisolone and placebo in females presenting with hyperemesis gravidarum during the first trimester of pregnancy.

While ensuring confidentiality, informed consent was taken and double blindness ensured. Follow-up after one week in clinic was done to rule out continuous vomiting (>1 times vomiting per day after one week of treatment) or high-frequency vomiting (>5 times vomiting per day still after one week of treatment).

Statistical Package for Social Sciences (SPSS) Statistics version 22 (IBM Corp., Armonk, NY, USA) was used to assess the data. Quantitative parameters (age, gestational age) and qualitative parameters (parity, continue vomiting, high-frequency vomiting) were assessed in terms of mean/standard deviation and frequency/percentage, respectively. Comparison of continue/high-frequency vomiting was done via chi-square test (significant P-value ≤ 0.05). Post-stratification chi-square test (significant P-value ≤0.05) was applied to confounding factors like age and parity.

## Results

Mean age in years, mean gestational age in weeks, and parity distribution <2 was almost equally distributed between the prednisolone group and placebo group as shown in Table 1 to allow reasonable comparison to be made between two groups. Frequency of vomiting (including continuous vomiting and high-frequency vomiting) was statistically significantly (P=0.002) reduced in patients taking prednisolone as compared to placebo, as shown in Table 2. Stratification of data with respect to age (Table 3) and parity (Table 4) of patients showed statistically insignificant differences between prednisolone and placebo groups.

**Table 1:** Distribution of mean age (years), mean gestational age (weeks) and parity < 2 amongst two groups

	Mean age	mean gestational age	parity of < 2
Prednisolone group	28.71±6.83	8.51±2.23	42 %
Placebo group	29.11±6.61	8.62±2.41	37. 4 %

**Table 2:** Distribution of vomiting (overall, continuous vomiting, high frequency)

	Frequency of vomiting	continuous vomiting	high frequency vomiting
Prednisolone group	28.7 %	18.7 %	10 %
Placebo group	46 %	20 %	26 %

**Table 3:** Age (years) stratification

	18-25 years		26-32 years		33-40 years	
	Prednisolone group	Placebo group	Prednisolone group	Placebo group	Prednisolone group	Placebo group
Vomiting	29 (54.7%)	24 (45.3%)	14 (28%)	18 (40.9%)	0 (0%)	27 (50.9%)
No vomiting	24 (45.3%)	29 (54.7%)	36(72%)	26 (59.1%)	47 (100%)	26 (49.1%)
P-value	0.331		0.188		0.000	

**Table 4:** Parity stratification

Parity	0		1		2		3		4	
Vomiting	Prednisolone group	Placebo group	Prednisolone group	Placebo group	Prednisolone group	Placebo group	Prednisolone group	Placebo group	Prednisolone group	Placebo group
Yes	18(56.3%)	15(48.4%)	12(38.7%)	11(44%)	11(22.9%)	23(46.9%)	2(7.4%)	15(51.7%)	0(0%)	5(31.3%)
No	14(43.8%)	16(51.6%)	19(61.3%)	14(56%)	37(77.1%)	26(53.1%)	25(92.6%)	14(48.3%)	12(100%)	11(68.8%)
P-value	0.532		0.689		0.013		0.033		0.000	

## Discussion

Although nausea and vomiting is commonly experienced by pregnant females, hyperemesis gravidarum is seen in only 2% cases [7,8]. Sometimes it might need fluid and electrolytes resuscitation on an emergency basis.

Different medications are available to treat hyperemesis gravidarum including antihistamine H1 receptor block­ers, phenothiazines, metoclopramide, Benzamine, ondansetron, vitamin B6 (pyri­doxine) +/- doxylamine (an antihistamine). When used in combination, around 70% reduction in symptoms is seen [8-10].

In this study it was observed that among women who were given prednisolone frequency of vomiting was lower than that of placebo group. i.e. Group A: 28.7% vs. Group B: 46%, P-value=0.002. Prednisolone effectively reduced the frequency of vomiting.

Except age group 18-25 years (P-value=0.331, Group A: 54.7% vs. Group B:45.3%) among women who were in the age group 26-40 years frequency of vomiting was lower in prednisolone group but it was only statistically significant in the age group 33-40 years (P-value=0.000).

Among women who were multiparous, a statistically significant difference was seen for vomiting in women who were given prednisolone. However among women who were nulli-parous and primary-parous frequency of vomiting was the same with prednisolone as with placebo.

In comparison, 16% of cases of steroid group had vomiting at one week versus 42% cases in non-steroid group in study conducted in Rawalpindi, Pakistan [11]. Results of this study are consistent with the findings of a local study showing that prednisolone is as effective for reducing frequency of vomiting as that of placebo.

Mardanian reported that the severity and the times of vomiting decreased and this improvement was seen more significantly in the prednisolone group (P-value< 0.05). His study findings also support the findings of this study that prednisolone is effective for reducing frequency of vomiting [12].

Safari et al. in his study gave prednisolone tablet 16 mg every eight hours for three days then gradually tapered for two weeks. The control group received promethazine tablets 25 mg every eight hours for two weeks. Results showed that prednisolone improved symptoms faster and shortened the period of fluid therapy, thus the duration of hospitalization decreased [13].

In a study by Piercy, patients were given prednisolone 20 mg every 12 hours intravenously for seven days while the control group received the same volume of placebo. The improvement in vomiting was seen in 80% of the cases in the study group and 30% of the control group [14].

Safari and Piercy also support the use of prednisolone for effective treatment against vomiting in women with hyperemesis gravidarum. 

Indications for the use of steroids in hyperemesis gravidarum are delineated. Most protocols suggest using steroids after the 10th week of pregnancy and limiting their use to one month. The initial dose is typically high, then weaned down slowly after a few days. Present study registered cessation of vomiting was observed in most of the patients after initial dose of intravenous hydrocortisone.

Maintenance prednisolone therapy permitted discharge from hospital within days, resumption of normal eating, reversal of muscle wasting and regain of lost weight mean loss from pre-pregnancy weight. Steroid use is typically saved for women who do not respond to other medications by the end of the first trimester, and are losing weight rapidly due to more severe nausea and vomiting. Use of steroids for hyperemesis gravidarum revealed benefits with respect of patients’ wellbeing, food intake and weight gain [15-17].

The mechanism of action by which corticosteroid therapy is able to reduce nausea and vomiting is unknown. In the prevention and treatment of corticosteroids induced nausea and vomiting, corticosteroids are employed in combination with antiemetics and have limited efficacy when used as monotherapy.

## Conclusions

Results of this study showed that corticosteroid is effective for treating women with hyperemesis gravidarum during the first trimester of pregnancy. Prednisolone reduces the frequency of continuous vomiting as well as high-frequency vomiting. Hence, it has significant effect in deceasing the frequency of vomiting overall during pregnancy. This effect is not related to the age or parity of the patient. Knowing the fact that prednisolone becomes inactive in the placenta and does not pass to the fetus makes its use safer during pregnancy.
